# Modeling and Numerical Investigation of Transient Two-Phase Flow with Liquid Phase Change in Porous Media

**DOI:** 10.3390/nano11010183

**Published:** 2021-01-13

**Authors:** Fei He, Wenjie Dong, Jianhua Wang

**Affiliations:** 1 CAS Key Laboratory of Mechanical Behavior and Design of Materials, Department of Thermal Science and Energy Engineering, University of Science and Technology of China, Jinzhai Road 96, Hefei 230026, China; hefeihe@ustc.edu.cn; 2 School of Optical Information and Energy Engineering, Wuhan Institute of Technology, Wuhan 430205, China; dwj105mc@mail.ustc.edu.cn

**Keywords:** two-phase flow in porous media, liquid phase change, transient behavior, heat transfer deterioration, vapor block

## Abstract

Two-phase flow with phase change in microstructure or nanostructure is an important issue in many fronts and critical applications nowadays, but with a lack of comprehensive understanding of the mechanism. This paper numerically investigates the transient behavior of two-phase flow with liquid phase change in the porous media, which consists of a series of connected pores at micro and nanoscale with the transient form of the semi-mixed model and self-compiled programs. Transient variation and spatial distribution of structure temperature, thermal non-equilibrium characteristic, phase change location and fluid-driven pressure are obtained and analyzed, and effects of initial system temperature, structure parameter and material property on the transient behaviors of two-phase flow and fluid-structure coupling heat transfer are discussed. The numerical simulations indicate that the two-phase flow with phase change in porous media is complex and ever-changing before reaching a steady state and affected by the above-mentioned three kinds of parameters significantly. Particularly, distinct phenomena of transient heat transfer deterioration and vapor block are discovered, and it is revealed that the transient heat transfer deterioration and vapor block are more serious in a porous matrix with smaller porosity and made of materials with higher heat capacity and density.

## 1. Introduction

Two-phase flow and heat transfer at the micro and nanoscale have attracted much attention from researchers in the fields of geothermal energy recovery [1,2], fuel cell technology [3,4], building energy conservation [5,6], mobile Internet devices [7,8,9], and spacecraft thermal protection [10,11]. In transpiration cooling, which is one of the most promising heat dissipation approaches of mobile Internet devices and spacecraft [9], by virtue of porous matrix with pores at micro and nanoscale, phase change coolant exchanges heat fully with structure by flowing through, keeping the whole structure at moderate temperatures. In proton exchange membrane fuel cell, one environment-friendly power source with high energy density, back-diffusion of water from cathode to anode always appears due to the differences in pressure and concentration during the operation of fuel cells, resulting in water flooding in the micropores of anode [4]. Therefore, the two-phase flow with phase change in microstructure or nanostructure is an important issue in many fronts and critical applications nowadays.

There is much research work on the steady problems of two-phase flow with liquid phase change in microstructure or nanostructure. A great number of experiments have been conducted to explore the wide range of two-phase flow regimes in different microstructures [12,13,14]. Several widely recognized mathematical models [15,16,17,18,19,20,21,22,23,24] and a series of numerical approaches have been developed to probe into the mechanism. Based on these models and numerical approaches, the thermal non-equilibrium characteristic between fluid and solid [25], and the influences of fluid mass flow rate of fluid [20,24], heat flux distribution [26,27], and geometry configuration [19] on the two-phase flow and heat transfer characteristics have been studied.

However, there is a lack of comprehensive analysis and knowledge about the transient behavior of two-phase flow with phase change in the porous media so far. Different from the general immiscible gas-liquid two-phase flow, for example, oil-gas flow in rock layers and cracks, when phase change of liquid fluid occurs in pores and channels at micro or nanoscale, there are significant mass, momentum and energy transfers between liquid phase and vapor phase, huge amount of latent heat is released, and capillary force is generated. The resultant dramatic variations of fluid density and specific volume affect the transport characteristic of the two-phase flow and the fluid-solid coupling heat transfer greatly. Therefore, affected by the micro or nanoscales of the connected pores and the distributions of heat and pressure at structure boundaries, some special thermal responses and fluid transport characteristics would arise during the transient process. The experimental data from Huang et al. [28] indicated that there was a temporal hysteresis in the response of structure temperature due to the mismatching of the sensitivity to the variations of external heat flux and internal fluid mass flow rate. This phenomenon was confirmed by Luan et al. [29], and they further discovered that under a certain thermal environment and a range of constant fluid injection ratios, both the structure temperature and the fluid pressure fluctuated dramatically. Overall, the two-phase flow with phase change in porous media goes through an initial stage with significant variation before reaching steady state, even when the thermal environment is stable. Therefore, it is very necessary and important to develop the transient mathematical model and investigate the dynamic evolution process of phase change and the transient behavior of fluid-structure coupling heat transfer in porous media.

In this paper, the transient form of the semi-mixed model under local thermal non-equilibrium condition is proposed to describe the transient two-phase flow with phase change in porous media, and a self-compiled program is developed as the numerical approach. Through the numerical simulation, the time-varying spatial distribution of important physical parameters, such as structure temperature, thermal non-equilibrium characteristic, phase change location and fluid-driven pressure are obtained and quantitatively analyzed, and the effects of system initial temperature, microstructure parameter and material property on the transient behaviors of two-phase flow and fluid-structure coupling heat transfer are systematically discussed.

## 2. Mathematical Model and Theoretical Analysis

**Figure 1:**
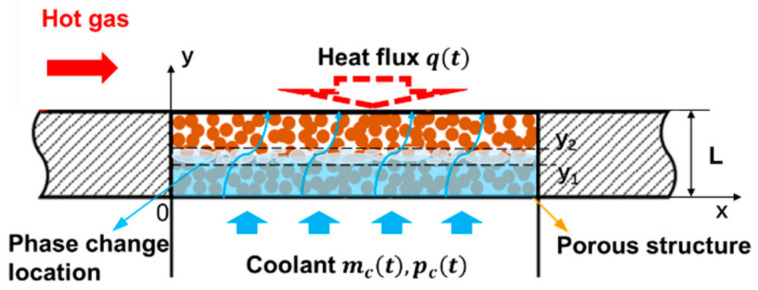
Physical model of transient two-phase flow with phase change in porous media.

Figure 1 shows the physical model for the transient problem of two-phase flow with phase change in porous media considered in this work. The homogeneous porous plate is located horizontally and has a thickness of *L*. Heat flux $q\left(t\right)$ is applied on the upper surface of the porous plate, while liquid fluid at temperature $T_{c}$ flows into the porous plate with a mass flow rate of $m_{c}\left(t\right)$ from the lower surface. The liquid fluid absorbs heat from the porous matrix up to phase change, and then vapor generates and flows out of the porous plate.

According to fluid states, there are three regions in the porous matrix from bottom to top: the liquid region, the mixed region of liquid and vapor phases, and the vapor region. Some mathematical models have been developed specifically to describe the characteristics of fluid flow and heat transfer in the three regions. Our previous research [16] has compared these models symmetrically, and the results indicated semi-mixed model (SMM) [19] was more accurate and flexible. Therefore, the transient form of SMM under local thermal non-equilibrium condition is proposed in this work to describe the transient two-phase flow with phase change in porous media.

### 2.1. Mass Conservation Equation

In the single-phase region, the mass conservation equation has uniform expression, using a subscript “i” to represent liquid phase “l” and vapor phase “v”:

(1) $$\frac{\partial }{\partial t}\left(\varepsilon \rho_{i}\right)=-\nabla \cdot \left(\rho_{i}\varepsilon u_{i}\right)$$

In the two-phase region, the mass conservation equations are separately described for liquid and vapor phases by liquid saturation s:

(2) $$\frac{\partial \varepsilon s\rho_{l}}{\partial t}+\nabla \cdot \left(\rho_{l}\varepsilon su_{l}\right)=-m^{\prime }$$

(3) $$\frac{\partial \varepsilon \rho_{v}\left(1-s\right)}{\partial t}+\nabla \cdot \left[\rho_{v}\varepsilon \left(1-s\right)u_{v}\right]=m^{\prime }$$

### 2.2. Momentum Conservation Equation

Similarly, the momentum conservation equation has uniform expression in the single-phase region, while it is separately described for liquid and vapor phases in the two-phase region.

In the single-phase region:

(4) $$\rho_{i}\frac{\partial u_{i}}{\partial t}=-\nabla p_{i}+\rho_{i}g-\frac{\mu_{i}}{K_{i}}\varepsilon u_{i}$$

In the two-phase region:

(5) $$\rho_{l}\frac{\partial u_{l}}{\partial t}=-\nabla p_{l}+\rho_{l}g-\frac{\mu_{l}}{K_{l}}\varepsilon su_{l}$$

(6) $$\rho_{v}\frac{\partial u_{v}}{\partial t}=-\nabla p_{v}+\rho_{v}g-\frac{\mu_{v}}{K_{v}}\varepsilon \left(1-s\right)u_{v}$$

### 2.3. Energy Conservation Equation

In the single-phase region, the conservation equation of fluid energy has uniform expression:

(7) $$\frac{\partial }{\partial t}\left(\varepsilon \rho_{i}H_{f}\right)=-\nabla \cdot \left(\frac{\varepsilon k_{i}}{c_{p,i}}\nabla H_{f}\right)+q_{sf}$$

In the two-phase region, the conservation equation of fluid energy is unitedly described for the mixture of liquid and vapor phases:

(8) $$\frac{\partial }{\partial t}\left\{\varepsilon \left[s\rho_{l}+\left(1-s\right)\rho_{v}\right]H_{f}\right\}+\nabla \cdot \left\{\varepsilon u_{v}\left[s\rho_{l}+\left(1-s\right)\rho_{v}\right]H_{f}\right\}+\nabla \cdot \left[\varepsilon \left(u_{l}-u_{v}\right)s\rho_{l}H_{l}\right]=\nabla \cdot \left(k_{f,eff}\nabla T_{f}\right)+q_{sf}$$

where $T_{f}$ is the temperature of the fluid, and $T_{l}=T_{v}=T_{f}$. $H_{f}$ is the specific enthalpy of the mixture of liquid and vapor phases, and its definition is:

(9) $$H_{f}=\frac{s\rho_{l}}{s\rho_{l}+\left(1-s\right)\rho_{v}}H_{l}+\frac{\left(1-s\right)\rho_{v}}{s\rho_{l}+\left(1-s\right)\rho_{v}}H_{v}$$

It can be found that when liquid saturation *s* = 1, fluid is in a liquid state and $H_{f}=H_{l}$; when 0<s<1, fluid is in a mixed state of liquid and vapor phases; when *s* = 0, fluid is in vapor state and $H_{f}=H_{v}$. Therefore $H_{f}$ is valid in all the single-phase and two-phase regions.

In all regions, energy conservation of a solid matrix is described by:

(10) $$\frac{\partial }{\partial t}\left[\left(1-\varepsilon \right)\rho_{s}c_{p,s}T_{s}\right]=\nabla \cdot \left(k_{s,eff}\nabla T_{s}\right)-q_{sf}$$

### 2.4. Constitutive Relations

After solving the mixed specific enthalpy, one can obtain the saturation and temperature of the fluid by the following algebraic relations:

(11) $$T=\left\{\begin{array}{cc} \left(H_{f}-H_{l,sat}\right)/c_{p,l}+T_{sat} & H_{f}\leq H_{l,sat}\\ T_{sat} & H_{l,sat}\leq H_{f}\leq H_{v,sat}\\ \left(H_{f}-H_{v,sat}\right)/c_{p,v}+T_{sat} & H_{v,sat}\leq H_{f} \end{array}\right.$$

(12) $$s=\left\{\begin{array}{cc} 1 & H_{f}\leq H_{l,sat}\\ \frac{\rho_{v}\left(H_{v,sat}-H_{f}\right)}{\rho_{l}\left(H_{f}-H_{l,sat}\right)+\rho_{v}\left(H_{v,sat}-H_{f}\right)} & H_{l,sat}\leq H_{f}\leq H_{v,sat}\\ 0 & H_{v,sat}\leq H_{f} \end{array}\right.$$

In the single-phase region, fluid in a vapor state is regarded as an ideal gas, while in the liquid state is considered to be incompressible. In the two-phase region, fluid is the mixture of liquid and vapor phases, of which the vapor phase maintains at saturation state, and the pressure of the vapor phase depends on the temperature:

(13) $$p_{v}=p_{0}\exp\left[\frac{h_{lv}}{R_{g}}\left(-\frac{1}{T}+\frac{1}{T_{0}}\right)\right]$$

Then the pressure of the liquid phase can be obtained by $p_{l}=p_{v}-p_{c}$, where $p_{c}$ is the capillary pressure.

Other constitutive relations used to close the solution are summarized in Table 1.

## 3. Numerical Approach

### 3.1. Initial and Boundary Conditions

The transient problem that a porous matrix full of liquid fluid begins to be exposed to a high heat flux is considered in this paper. Hence, the initial conditions for the whole physical domain are set as:

$${\left.T_{s}\left(x,t\right)\right|}_{t=0}=T_{s}^{0}\;{\left.T_{f}\left(x,t\right)\right|}_{t=0}=T_{f}^{0}\;{\left.s\left(x,t\right)\right|}_{t=0}=1$$

According to the discussion results in previous research [16], the most reasonable and feasible boundary conditions at the hot side and cold side are selected and employed in this work.

At cold side:

$$\rho_{l}\varepsilon v_{l}=m\left(t\right)\;h_{c}\left(T_{s}-T_{c}\right)=k_{s,eff}\frac{dT_{s}}{dy}\;h_{c}\left(T_{s}-T_{c}\right)=m\left(t\right)\left(H_{f}-H_{l}\left(T_{c}\right)\right)$$

At hot side:

$$p=p_{0}\left(t\right)\;k_{s,eff}\frac{dT_{s}}{dy}=q\left(t\right)\;\frac{dH_{f}}{dy}=0$$

### 3.2. Solution Procedure

The transient SMM under local thermal non-equilibrium condition is solved by a self-compiled program at University of Science and Technology of China. The finite difference method is employed to discretize the governing equations: explicit scheme [30,31] is used for the time term; second-order upwind difference, central difference and linearized schemes are, respectively, used for the convection term, the diffusion term and the source term in the energy equations. After the discretization, a set of linear algebraic equations are obtained and then solved by an iterative algorithm. The discretized equations of energy, mass and momentum are solved sequentially at each time step, after achieving the convergence, the solving procedure enters the next time step. Convergence is judged by the same criteria: the relative changes of $T_{s}$, $T_{f}$ and *p* between successive iterations are less than $10^{-6}$. Liquid water is considered as the fluid, and its thermal properties are listed in Table 2. Table 3 presents the reference values of the structure parameters and the thermal properties of the porous matrix used in the simulations.

## 4. Experiment and Verification

**Figure 2:**
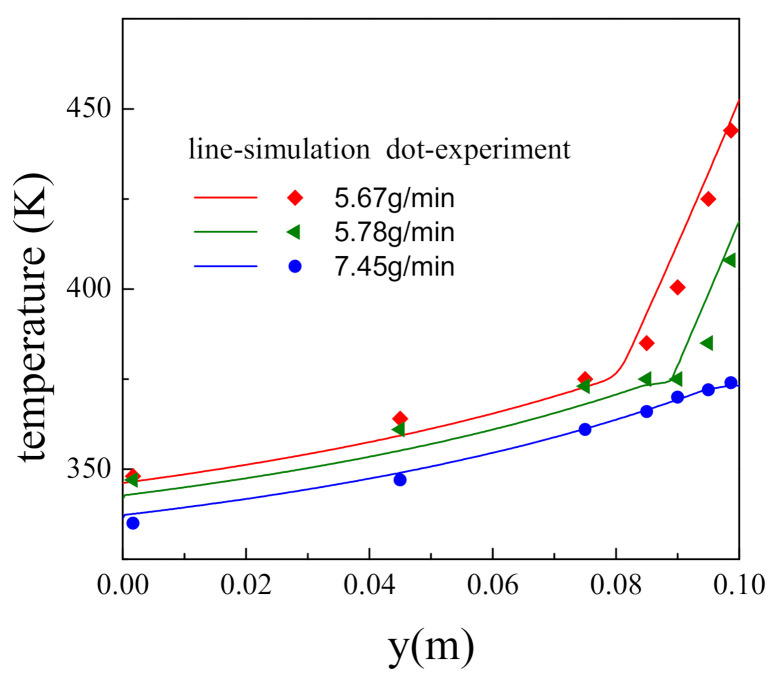
Comparison of numerical results and experimental data.

Due to the lack of proper transient experimental data in open literature, the mathematical model and numerical approach proposed in this work are validated with the steady-state experimental data from Hu et al. [32]. In the experiment, the porous cylindrical test section was consisted of sintered bronze with a porosity of 0.318 and a particle diameter of 0.2 mm and had a diameter of 40 mm and a height of 100 mm. An average heat flux 0.21 MW/m^2^ was applied to the upper surface of the porous test section, and liquid water with an initial temperature of 300 K was injected from the lower surface. The wall temperature along the flow direction was measured. Simulations under the completely identical operating condition with the experiments were carried out with our mathematical model and numerical approach, and the comparison results are shown in Figure 2. The maximum relative deviation between the experimental and simulation results is less than 4%, which demonstrates the mathematical model and numerical approach suggested are valid to a certain extent.

## 5. Results and Discussions

### 5.1. Transient Behavior of Two-Phase Flow with Phase Change in Porous Media

**Figure 3:**
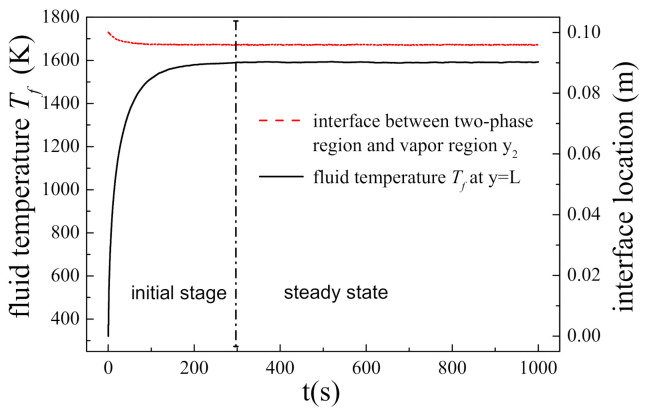
The transient variations of fluid temperature at the hot side and phase interface position y_2_.

The two-phase flow with phase change in porous media will eventually reach a steady state when the thermal environment and fluid inject rate are unchanged. However, before that, the temperature of the porous structure, the location of phase change and the driven pressure of fluid change acutely with time, and this stage are known as initial stage. Figure 3 shows the transient behavior of the porous system with an initial temperature of 300 K when subjected to a constant heat flux of 1.5 × 10^6^ W/m^2^ and cooled by liquid water with a mass flow rate of 0.3 kg/m^2^s. It can be found that the fluid temperature at the hot side and the location of the interface between two-phase region and vapor region approach certain values at approximately 300 s and then maintain unchanged, which means the whole system reach the steady state by this time. From the beginning t = 0 s to t = 300 s, the system goes through an initial stage, in which physical parameters vary with time significantly.

**Figure 4:**
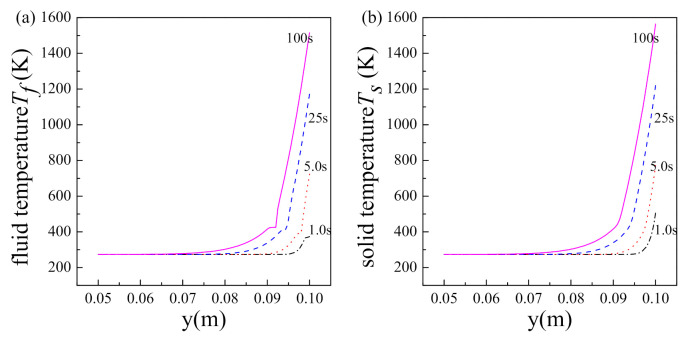

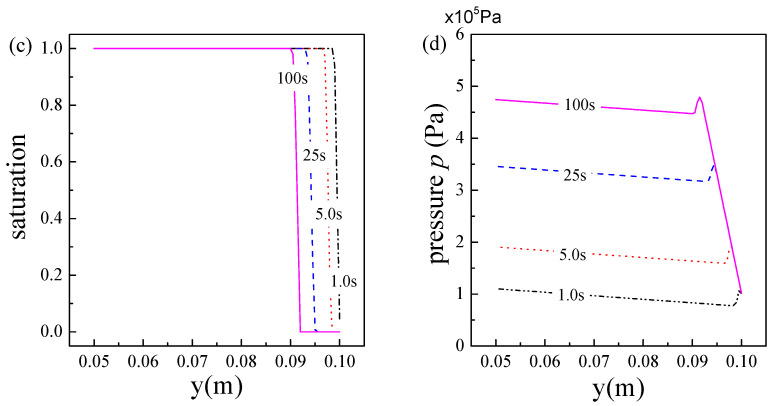
The distributions of (**a**) solid temperature, (**b**) fluid temperature, (**c**) fluid saturation and (**d**) fluid pressure within porous matrix in the initial stage.

To study the two-phase fluid flow and fluid-solid heat transfer characteristics in the initial stage, Figure 4 exhibits the distributions of the temperature, pressure and saturation of fluid within porous matrix at t = 1, 5, 25 and 100 s. As shown in Figure 4, the spatial distributions of fluid temperature and pressure have same variation trends at different times: the fluid temperature rises slowly in the liquid region, keeps almost constant in the two-phase region, and increases sharply in the vapor region; the pressure drops gradually in the liquid region, rises rapidly in the two-phase region, and decreases substantially in vapor region. Conforming to the same flow and heat transfer mechanisms, the spatial distribution laws of physical parameters obtained at different times in the initial stage are reasonable, and identical with those in the steady state obtained by steady SMM [16].

Combining Figure 3; Figure 4, it could be found that: the porous structure is full of liquid water at first, and the temperature and pressure of fluid are low; as time goes by, phase change occurs, the location of phase change moves down and vapor generates, and then the temperature and pressure of fluid increase monotonically; finally, there are three regions, i.e., vapor region, two-phase region and liquid region, sequentially formed within the porous structure. We find that the maximum temperature and pressure always appear at either the hot side or cold side. Moreover, the maximum temperature and fluid-driven pressure of the two-phase flow with phase change in porous media are most concerned in engineering applications. Therefore, we focus on the transient variation of physical parameters on the boundary in the following study.

### 5.2. Effect of Initial Temperature

**Figure 5:**
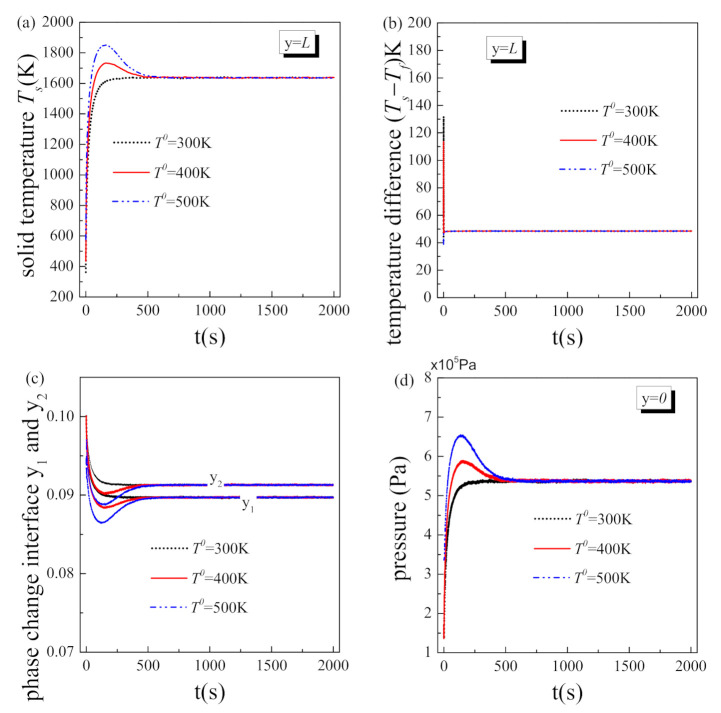
Transient results under different initial temperatures: (**a**) solid temperature at the hot side; (**b**) temperature difference at the hot side; (**c**) phase-change location; (**d**) fluid pressure at inlet.

Figure 5 exhibits the transient variation of the physical parameters on the boundary obtained under different system initial temperatures when other operating parameters remain to be the same with the reference value in Table 3. As shown in Figure 5, the solid temperature at the hot side always tends to the same value when the whole system reaches steady state, regardless of the initial temperature. Similar numerical results can be obtained for the temperature difference between solid and fluid at the hot side, phase change location and fluid pressure at inlet. This is reasonable, because the steady state values of the physical parameters depend on the operation condition rather than the initial condition. For the porous matrix with fixed microstructure, when the heat flux on the boundary and the fluid mass flow rate both keep unchanged, the heat transfer between the solid matrix and the fluid will eventually achieve same equilibrium, although may spend different length of time. As the results, the spatial distributions of all the physical parameters display same values at the equilibrium or steady state.

However, the initial temperature of the porous system has a great influence on the transient variation of the physical parameters in the initial stage. As shown in Figure 5a, the solid temperature presents quite different variation trends in the initial stage when the system initial temperature is different. The solid temperature increases to the steady value with a low initial temperature of 300 K while briefly exceeds the steady value in the initial stage with higher initial temperatures of 400 K and 500 K. These results are very important because the temperature of the solid matrix is usually concerned most by the engineers and designers in the industry. Supposing that the melting point of the porous material is 1400 °C the steady solid temperatures obtained under the three initial conditions are the same and lower than this melting temperature. This indicates the fluid amount applied could ensure no ablation in the steady state under the operation condition considered here. However, when the system initial temperature is 400 K and 500 K, the temperature of the solid matrix has exceeded the melting point before reaching the steady state, i.e., ablation has already occurred; only when the system initial temperature is 300 K, the temperature of the solid matrix is always lower than the melting point. This is a distinct phenomenon of transient heat transfer deterioration, which alerts us that: it is not enough to only ensure the steady temperature of the whole system lower than the critical value, because ablation may occur before the system reaches steady state. Therefore, the coolant injection amount should be increased in the initial stage for the case that the system initial temperature is relatively high.

Figure 5b,c depict the effect of system initial temperature on the transient variation of phase interface location within porous structure and thermal non-equilibrium characteristic at the hot side. The thermal non-equilibrium characteristic is greatly affected by the magnitude of phase change and the phase distribution of fluid. According to the discussion in steady state [16], the temperature difference between solid and fluid increases rapidly at the beginning of the two-phase region and reaches the maximum at the interface between the two-phase and vapor regions. Therefore, the thermal non-equilibrium characteristic at the hot side is particularly significant at the beginning of the transient process of the two-phase flow with liquid phase change in porous media with an initial temperature of 300 K. When the porous structure is subjected to high heat flux, phase change briefly appears on the boundary. Solid temperature at the hot side increases rapidly, while fluid temperature there almost keeps constant due to latent heat, resulting in a locally remarkable temperature difference. When the system initial temperature is 400 K, the time of fluid phase change on the boundary shortens, and consequentially the thermal non-equilibrium characteristic there is not so strong. As the system, initial temperature increases to 500 K, fluid has no time to flow to the boundary and phase change occurs midway, so the thermal non-equilibrium characteristic at the hot side is weak.

The magnitude of phase change and phase distribution of fluid also has great influence on the driven pressure of the fluid. From Figure 5c, it could be found that: when *T^0^* = 300 K, the area of vapor region in the initial stage is always smaller than that in the steady state, but when *T^0^* = 400 K and 500 K, the area of vapor region in the initial stage expands quickly and once exceeds the steady value. For the flow resistance of vapor is much higher than that of liquid, larger vapor region inevitably corresponds to larger fluid pressure at inlet. As shown in Figure 5d, when *T^0^* = 400 K and 500 K, the fluid pressure at inlet in the initial stage likewise exceeds the steady value. This is a distinct phenomenon of transient vapor block, which is worthy of attention. The fluid-driven pressure is an extremely important parameter in the design of a two-phase flow system and is usually set according to steady-state operating conditions. However, based on the results obtained here, the driven force of fluid required in the initial stage could actually be much greater than that in the steady state. If lacking driven force in the initial stage, the fluid supply must break down, so in this situation, the cooling system may already fail in the initial stage.

### 5.3. Effect of Porosity

**Figure 6:**
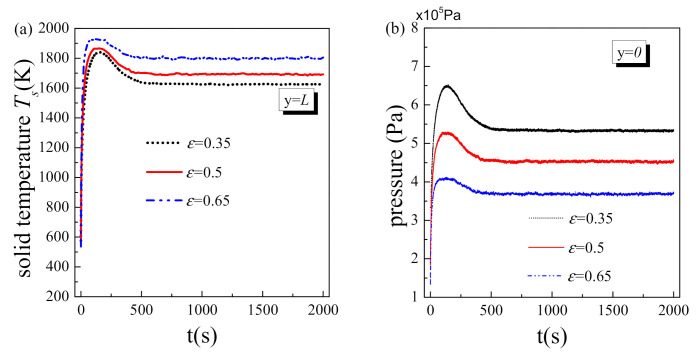
Transient results at different porosity: (**a**) solid temperature at the hot side; (**b**) fluid pressure at inlet.

Figure 6 exhibits the effect of porosity on the transient variations of solid temperature at the hot side and fluid pressure at inlet. The system initial temperature is set as 500 K to embody the distinct phenomena of transient heat transfer deterioration and vapor block. From Figure 6a, the maximum of solid temperature at the hot side could be observed in the initial stage, i.e., transient heat transfer deterioration, which is consistent with the result in Figure 5. With the increase of porosity, the solid temperature at the hot side increases while the transient heat transfer deterioration reduces. As listed in Table 1, the specific surface of a pore is linear function of the porosity, i.e., *α* = 6(1−*ε*)/*d_p_*, which indicates that fluid-to-solid heat transfer coefficient decreases with the porosity. Hence, more heat is stored in the solid matrix with larger porosity, resulting in a higher solid temperature. From Figure 6b, it could be found that the fluid pressure at inlet and the transient vapor block effect both decrease with the porosity. This is because solid matrix with larger porosity has smaller effective thermal conductivity *k_s.eff_*. As the results, the temperature gradient within the solid matrix becomes larger and vapor region becomes smaller. Accordingly, the resistance of fluid flowing through the vapor region reduces, i.e., the driven pressure decreases. It should be particularly noted that the porosity not only affects the transient variation of physical parameters in the initial stage but also changes their steady values. Large porosity design could greatly reduce the fluid-driven force and impair the transient heat transfer deterioration and vapor block, but at the cost of increasing the system temperature. Therefore, porosity is an important structure parameter of the two-phase flow with liquid phase change in porous media and should be given special consideration in the practice of engineering design.

### 5.4. Effect of Material Properties

**Figure 7:**
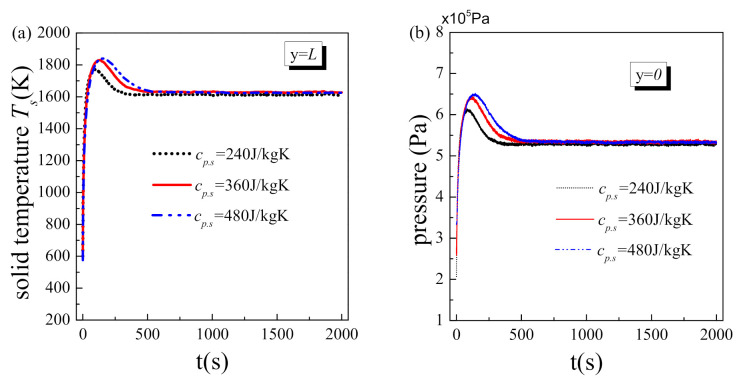
Transient results at different material heat capacity: (**a**) solid temperature at the hot side; (**b**) fluid pressure at inlet.

Figure 7 shows the effect of material heat capacity on the transient variations of solid temperature at the hot side and fluid pressure at inlet. The system’s initial temperature is 500 K, while other operating parameters remain to be the same with the reference values in Table 3. As the heat capacity of the solid matrix increases, the system spends more time reaching steady state, and the transient heat transfer deterioration in the initial stage is severer. This is reasonable because the larger the heat capacity of the solid matrix, the slower the variation of solid temperature, and accordingly, the longer the time required by the system to reach a steady state. On the other hand, solid matrix with larger heat capacity stores more heat, which is hard to release in a short time and inevitably causes severer transient heat transfer deterioration. The maximum fluid pressure in Figure 7b shows the same variation trend as that of solid temperature in Figure 7a, i.e., the transient vapor block effect become more significant with the increase of material heat capacity. After all, a higher solid temperature on the hot side corresponds to a larger vapor region and thereby greater flow resistance. As a result, the fluid pressure at inlet is higher.

**Figure 8:**
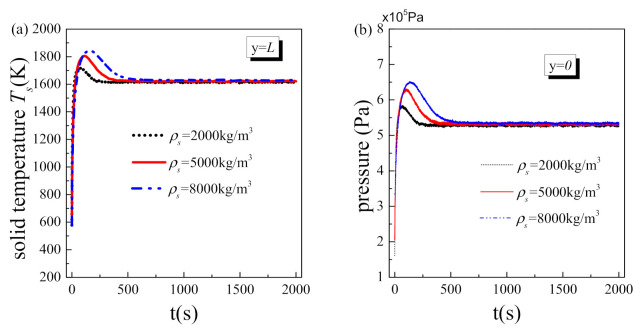
Transient results at different solid density: (**a**) solid temperature at the hot side; (**b**) fluid pressure at inlet.

Figure 8 shows the effect of material density on the transient variations of solid temperature at the hot side and fluid pressure at inlet. For the similar reason, as the density of the solid matrix increases, the system spends more time reaching steady state, and the transient heat transfer deterioration and the transient vapor block effect in the initial stage both become more significant. Overall, material properties have significant influence on the initial stage of the two-phase flow with liquid phase change in porous media, but no influence on the steady value of structure temperature and fluid-driven pressure. Particularly, the transient heat transfer deterioration and the transient vapor block effect are reduced when a material with low heat capacity and density is employed. Therefore, this provides an approach for the engineer and designer to impair the transient heat transfer deterioration and vapor block and avoid the corresponding negative effects.

## 6. Conclusions

This work is aimed at providing a comprehensive understanding of transient two-phase flow with a liquid phase change at the micro and nanoscale. A new mathematical model and corresponding numerical approach are developed, through which the dynamic evolution of phase change and transient behavior of fluid-structure coupling heat transfer are analyzed and the influences of initial system temperature, structure parameter and material property are discussed. From the simulations and analysis, the following conclusions can be drawn:

(1) For the porous matrix with fixed microstructure, when the heat flux on the boundary and the fluid mass flow rate both keep unchanged, the two-phase flow with liquid phase change in porous media will eventually achieve equilibrium, which is known as the steady state. Before reaching steady state, the transient two-phase flow with liquid phase change in porous media goes through an initial stage, in which the physical parameters vary with time significantly;
(2) The system initial temperature has no influence on the steady state of the two-phase flow with liquid phase change in porous media. However, when the initial system temperature is relatively high, distinct transient heat transfer deterioration and vapor block occur in the initial stage, which may cause unexpectedly premature cooling failure and structure ablation;
(3) The porosity not only affects the transient variation of the physical parameter in the initial stage but also changes their steady values. Large porosity design could greatly reduce the fluid-driven force and impair the transient heat transfer deterioration and vapor block, but at the cost of increasing the system temperature;
(4) As the heat capacity and the density of the solid material increases, the system spends more time reaching a steady state, and the transient heat transfer deterioration and vapor block effect in the initial stage are severer. Therefore, porous matrix made of a material with low heat capacity and density could reduce the transient heat transfer deterioration and the transient vapor block effect without affecting the steady state.

## Figures and Tables

**Table 1:** Constitutive relations.

Capillary pressure	$p_{c}=p_{v}-p_{l}={\left(\frac{\varepsilon }{K}\right)}^{1/2}\sigma J\left(s\right)$ $J\left(s\right)=1.417\left(1-s\right)-2.120{\left(1-s\right)}^{2}+1.263{\left(1-s\right)}^{3}$
Relative permeability	$K_{l}=s^{3}\;\;\;K_{v}={\left(1-s\right)}^{3}$
Effective thermal conductivity	$k_{s,eff}=\left(1-\varepsilon \right)k_{s}\;\;\;k_{f,eff}=\varepsilon sk_{l}+\varepsilon \left(1-s\right)k_{v}$
Fluid-to-solid heat transfer coefficient	$h_{si}=\left(k_{i}/d_{p}\right)\left(2.0+1.1Pr_{i}^{1/3}Re_{p}^{0.6}\right)\;\;\;i=l,v$
Specific surface	$\alpha =6\left(1-\varepsilon \right)/d_{p}$
Convective heat transfer of fluid-to-solid in pores	In the single-phase regions: $q_{sf}=h_{si}\alpha \left(T_{s}-T_{f}\right)\;\;\;i=l,v$ In two-phase regions: $q_{sf}=q_{boil}+\left(1-s\right)h_{sv}\alpha \left(T_{s}-T_{f}\right)$$q_{boil}=s\alpha \mu H_{lv}{\left[\frac{g\left(\rho_{l}-\rho_{v}\right)}{\sigma }\right]}^{1/2}{\left[\frac{c_{pf}\left(T_{s}-T_{sat}\right)}{0.006H_{lv}}\right]}^{1/0.33}{\left(\frac{1}{Pr_{f}}\right)}^{1.7/0.33}$
Specific enthalpy	$H_{i}=c_{p,i}\left(T-T_{0}\right)+H_{i,0}\;\;\;i=l,v$

**Table 2:** Thermal properties of water (Xin [23]).

Property (Units)	Liquid	Vapor
Density (kg∙m^−3^)	957.85	Ideal gas law
Specific heat (J∙kg^−1^∙K^−1^)	4217	2029
Thermal conductivity (10^−3^ W∙m^−1^∙K^−1^)	680	−21.994433+0.11842T
Prandtl number	$\mu_{l}c_{p}/k_{l}$	0.984
Dynamic viscosity (10^−6^ N∙s∙m^−2^)	$24.14\times 10^{\frac{247.8}{T-140}}$	−2.77567+0.04035T
Specific enthalpy at *T*_0_ = 373.15 K, *p*_0_ = 1 atm(10^6^ J∙kg^−1^)	2.676	0.419
Latent heat (10^6^ J∙kg^−1^)	2.257	

**Table 3:** Reference value of parameters used in simulations.

Heat flux at the hot side (W m^−2^)	*q* = 1.5 × 10^6^
Liquid water mass flow rate (kg m^−2^ s^−1^)	*m* = 0.3
Thickness of porous media (m)	*L* = 0.1
Porosity	*ε* = 0.35
Thermal conductivity of solid (W m^−1^ K^−1^)	*k_s_* = 13.4
Particle diameter (m)	*d_p_* = 5 × 10^−5^
Heat transfer coefficient at the cold side (W m^−2^ K^−1^)	*h_c_* = 31.4
Liquid water temperature at the cold side (K)	*T_c_* = 300

## Data Availability

The data presented in this study are available on request from the corresponding author.

## Nomenclature

*c_p_*	specific heat, J∙kg^−1^∙K^−1^
*d_p_*	particle diameter, m
**g**	gravity vector
*h*	heat transfer coefficient, W∙m^−2^∙K^−1^; or specific enthalpy, J∙kg^−1^
*h_lv_*	latent heat of evaporation, J∙kg^−1^
*K*	permeability, m^2^
*k*	thermal conductivity, W∙m^−1^∙K^−1^
*L*	length of the porous matrix, m
*m*	mass flow rate per unit area, kg∙m^−2^∙s^−1^
*m’*	interfacial mass transfer rate, kg∙m^−3^∙s^−1^
*p*	pressure, Pa
*q*	heat flux, W∙m^−2^
*R_g_*	gas constant of air, J∙kg^−1^∙K^−1^
*s*	liquid saturation
*T*	temperature, K
**u**	velocity vector
*u*, v	velocity components along x and y axes, m/s
*x*, *y*	Cartesian coordinate, m
**Greek Symbols**	
*𝜀*	porosity
*μ*	dynamic viscosity, N∙s∙m^−2^
*ρ*	density, kg∙m^−3^
σ	interfacial tension, N∙m^−1^
**Subscripts**	
0	reference
*c*	coolant
*eff*	effective
*i, f*	fluid in a different region
*l, v*	liquid, vapor
*s*	solid
*sat*	saturated state
*sf*	fluid to solid
**Superscripts**	
0	initial
